# A comparison of machine learning models for predicting urinary incontinence in men with localized prostate cancer

**DOI:** 10.3389/fonc.2023.1168219

**Published:** 2023-04-12

**Authors:** Hajar Hasannejadasl, Biche Osong, Inigo Bermejo, Henk van der Poel, Ben Vanneste, Joep van Roermund, Katja Aben, Zhen Zhang, Lambertus Kiemeney, Inge Van Oort, Renee Verwey, Laura Hochstenbach, Esther Bloemen, Andre Dekker, Rianne R. R. Fijten

**Affiliations:** ^1^ Department of Radiation Oncology (MAASTRO), GROW School for Oncology and Reproduction, Maastricht University Medical Center, Maastricht, Netherlands; ^2^ Department of Urology, Netherlands Cancer Institute, Amsterdam, and Amsterdam University Medical Centers, Amsterdam, Netherlands; ^3^ Department of Human Structure and Repair, Department of Radiation Oncology, Ghent University Hospital, Ghent, Belgium; ^4^ Department of Urology, Maastricht University Medical Center, Maastricht, Netherlands; ^5^ Department of Research and Development, Netherlands Comprehensive Cancer Organization, Utrecht, Netherlands; ^6^ Radboud Institute for Health Sciences, Radboud University Medical Center, Nijmegen, Netherlands; ^7^ Department of Urology, Radboud University Medical Center, Nijmegen, Netherlands; ^8^ Center of Expertise for Innovative Care and Technology (EIZT), School of Nursing, Zuyd University of Applied Sciences, Heerlen, Netherlands; ^9^ Expertise Center Empowering Healthy Behavior, Fontys University of Applied Sciences, Eindhoven, Netherlands

**Keywords:** prostate cancer, personalized medicine, machine learning (ML), PROMs = patient-reported outcome measures, urinary in continence, prediction modeling, shared decision making

## Abstract

**Introduction:**

Urinary incontinence (UI) is a common side effect of prostate cancer treatment, but in clinical practice, it is difficult to predict. Machine learning (ML) models have shown promising results in predicting outcomes, yet the lack of transparency in complex models known as “black-box” has made clinicians wary of relying on them in sensitive decisions. Therefore, finding a balance between accuracy and explainability is crucial for the implementation of ML models. The aim of this study was to employ three different ML classifiers to predict the probability of experiencing UI in men with localized prostate cancer 1-year and 2-year after treatment and compare their accuracy and explainability.

**Methods:**

We used the ProZIB dataset from the Netherlands Comprehensive Cancer Organization (Integraal Kankercentrum Nederland; IKNL) which contained clinical, demographic, and PROM data of 964 patients from 65 Dutch hospitals. Logistic Regression (LR), Random Forest (RF), and Support Vector Machine (SVM) algorithms were applied to predict (in)continence after prostate cancer treatment.

**Results:**

All models have been externally validated according to the TRIPOD Type 3 guidelines and their performance was assessed by accuracy, sensitivity, specificity, and AUC. While all three models demonstrated similar performance, LR showed slightly better accuracy than RF and SVM in predicting the risk of UI one year after prostate cancer treatment, achieving an accuracy of 0.75, a sensitivity of 0.82, and an AUC of 0.79. All models for the 2-year outcome performed poorly in the validation set, with an accuracy of 0.6 for LR, 0.65 for RF, and 0.54 for SVM.

**Conclusion:**

The outcomes of our study demonstrate the promise of using non-black box models, such as LR, to assist clinicians in recognizing high-risk patients and making informed treatment choices. The coefficients of the LR model show the importance of each feature in predicting results, and the generated nomogram provides an accessible illustration of how each feature impacts the predicted outcome. Additionally, the model’s simplicity and interpretability make it a more appropriate option in scenarios where comprehending the model’s predictions is essential.

## Introduction

Oncology is transitioning to more personalized and patient-centered care through interventions like data-driven prediction modeling and shared decision-making (SDM) (1). Of particular interest are patients for whom the possible treatment options are not superior to each other, and thus consideration of patients’ preferences and values plays an important role (2). This is the case for many patients with localized prostate cancer. Prostate cancer is one of the most common cancers among men with an incidence of 1,414,000 worldwide and 14,580 in the Netherlands in 2020 (3). Quality of life (QoL) of patients with prostate cancer can be adversely affected in a number of ways, such as through sexual, urinary, and bowel problems in the shorter or longer term (4). As a result, patients newly diagnosed with localized prostate cancer and their doctors face the challenge of choosing the appropriate treatment option from the main options including radical prostatectomy (RP), brachytherapy (BT), external beam radiotherapy (EBRT), and active surveillance (AS) (5). Though their choice will not negatively affect their survival (6), it is still a challenging decision due to the trade-off between harms and benefits associated with their decision i.e. risk of side effects such as urinary incontinence (UI) (7).

To reduce uncertainty around treatment decision-making, providing data-driven insights into outcomes such as UI is crucial. Therefore, machine learning (ML) algorithms can be applied to datasets to predict an individual’s risk, ultimately assisting patients and clinicians in treatment decision-making (8). In healthcare, logistic regression is referred to as an explainable model because of its formulaic nature (9). However, some research questions require more complex algorithms to achieve desired accuracy levels, but may suffer from a lack of transparency, and therefore a lack of clinical adoption (10). Maintaining a balance between accuracy and explainability in machine learning models is crucial, but previous studies have hypothesized that higher accuracy may lead to a reduced level of explainability, which is referred to as black-box algorithms (11). Few studies used ML methods to predict the development of UI after treatment, which makes it difficult to conclude whether there is an association between the accuracy and explainability of models on the outcome. For example, Park et al. (12) compared a series of models and found that the black box models were superior compared to the logistic regression model. The primary objective of the current study was to develop models to predict the risk of developing UI 1-year and 2-years post-diagnosis in men with localized prostate cancer and identify the important predictors. The secondary aim of our study was to evaluate the potential of three different ML algorithms for predicting UI risk in this patient population. Comparing performance of these models provides insights into the potential benefits of using simpler models in scenarios like predicting UI risk, where transparency and interpretability are important, and where error is costly.

## Methods

### Study population

In this study we used a subset of the Prostaatkanker Zorg in Beeld (ProZIB) dataset. The ProZIB data collection was embedded in the framework of the Netherlands Cancer Registry held by the Netherlands Comprehensive Cancer Organisation (IKNL). All clinical data was retrieved by well-trained data managers of IKNL. PROMs data were collected by several questionnaires, including the EPIC-26 questionnaire (13).

The subset of ProZIB used for the current study included 964 patients with T1-T3a prostate cancer diagnosed and treated in one or more of 65 Dutch hospitals with data available on a wide range of demographic, clinical, and PROMs variables at diagnosis and 1 year and 2 years after diagnosis. The details of this ProZIB subset and the population’s characteristics were previously described (14).

### Data cleaning and analysis

Data cleaning was performed similarly to our previous study that examined erectile dysfunction (15). Briefly, the outcome was based on a dichotomous transformation of the answers to question 1 in the EPIC-26 questionnaire (13). In this question patients were asked to respond to the following: “Over the past 4 weeks, how often have you leaked urine?” On a scale of 1 to 5, patients assessed their condition.

While 5 represented “rarely or never” experiencing UI problems, 1 to 4 indicated the presence of UI problems ranging from severe to mild. In this binary transformation patients who answered 5 (rarely or never) were grouped in one group and other answers (1-4 were) in the other group.

To predict patient outcomes, clinical factors and baseline PROMs were analyzed alongside the chosen treatment category for each patient. Missing data were dealt with by excluding variables with high rates of missing data and by removing patients with excessive missing values (i.e., above the 95th percentile). The 2-year time point contained a greater number of missing values due to loss of follow-up, so one dataset for each outcome time point was created: a 1-year dataset and a 2-year dataset. Datasets for the first and second years contained 848 and 670 patients, respectively. A detailed explanation of the pre-processing steps can be found in Hasannejadasl et al. (15). To perform TRIPOD (Transparent reporting of a multivariable prediction model for individual prognosis or diagnosis) Type 3 validation, it is necessary to externally validate data from a different location or time. For the purpose of external validation in this study, we divided the data by hospital, so that the dataset from each hospital was either used to train or validate (614 train data for 1-year and 479 for 2-year).

Our preliminary findings revealed that our dataset exhibited class imbalance following the data preprocessing procedures outlined in (15). To address this issue, we performed upsampling of the minority class to resolve the imbalance in the number of samples between the two classes using Synthetic Minority Oversampling Technique (SMOTE) (16). SMOTE is an upsampling method that mathematically generates synthetic data samples based on the data distribution of the real data. The SMOTE algorithm generated 214 and 175 synthetic data training samples for the 1-year and 2-year respectively, resulting in a total of 828 and 654 samples for each dataset respectively.

### Machine learning

We used three ML algorithms - Logistic Regression (LR), Random Forest (RF), and Support Vector Machines (SVM) - for both time points. We conducted recursive feature selection on all 36 variables, and then evaluated each feature’s relationship with the outcome to choose the most pertinent features. For LR and SVM models, we applied bootstrapping using the “glmnet” (17) and “caret” (18) packages, respectively, while for RF models, we utilized cross-validation in R version 3.6.3.

After developing the prediction models, we generated Receiver Operating Curves (ROCs) using the “pROC” package (19) in R and calculated the sensitivity, specificity, and overall accuracy. We used the variance inflation factor (VIF) to assess the degree of multicollinearity among the predictors in the LR model (20). To assess the agreement between predicted probabilities and observed frequencies in the test set, calibration plots were employed using the tidyverse package (21) in R. We utilized the coefficients from LR models to demonstrate the significance of each predictor, and performed feature importance analysis on the predictors of RF and SVM models using the Permutation Importance principle. Finally, to provide a visual representation of the 1-year LR model, we created a nomogram using the “rms” package in R (22).

## Results

### Population characteristics

The characteristics of the patient population in our cohort were described previously (15). Briefly, N0 stage was registered in 57.9% of patients; the remainder was defined as NX (lymph nodes were not investigated for the presence of cancer cells). The median age of participants was 68 years, while the majority of them (73%) were in the 60-75 age group. 41.3% of patients in the 1-year dataset and 40.3% of patients in the 2-year dataset received no active therapy, and the number of patients who underwent EBRT was relatively small (6.1% and 6.3% for the two time points respectively). The variables used as input for the prediction models for both years are shown in details in **Supplementary Table 1**. The degree of multicollinearity among the predictors was evaluated using the VIF, which revealed that all predictor values were relatively low. As a result, we concluded that multicollinearity did not pose a significant problem in our analysis (see **Supplementary Table 2** in the Supplementary Material for details).

**Figure 1:**
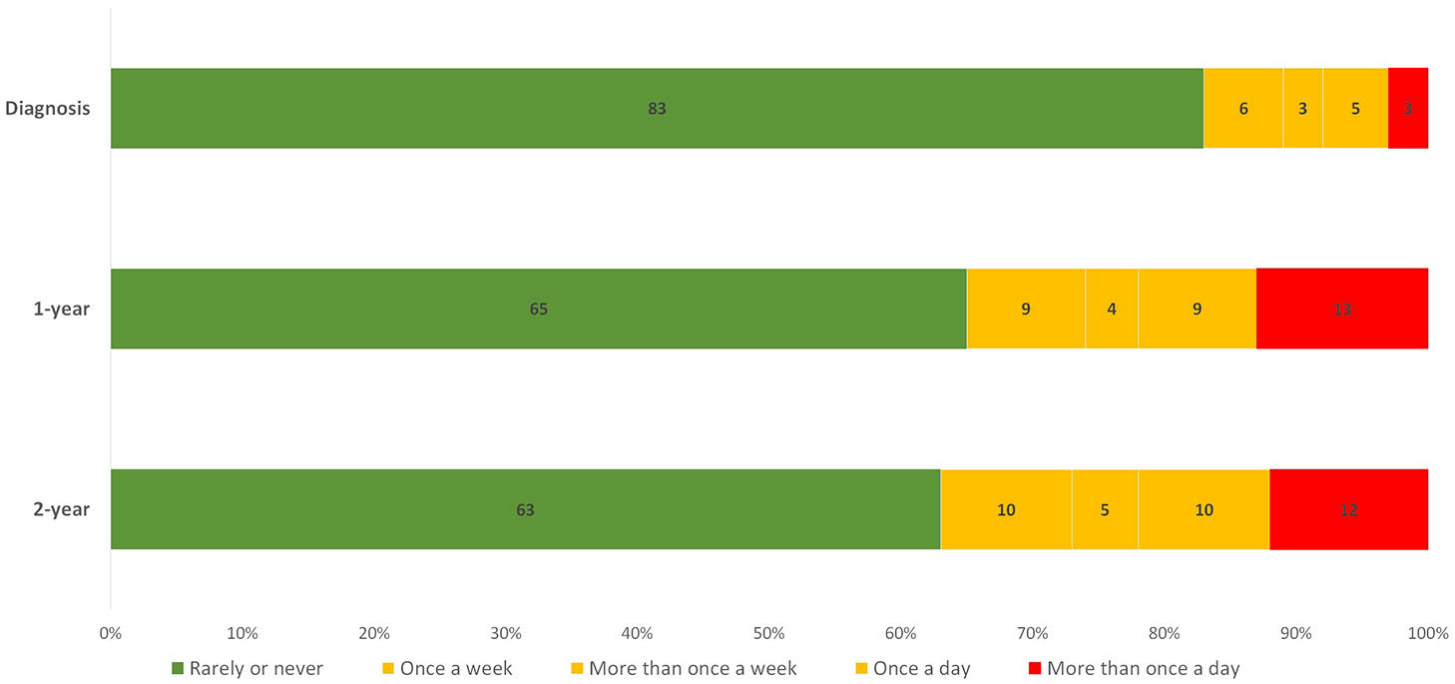
Distribution of the UI answers given by patients at diagnosis, 1-year and 2-year. The numbers displayed inside each bar are the percentages of the total number of patients for each individual time point. Green, yellow, and red bars represent patients with the least, moderate, and most severe forms of UI problems, respectively.

In addition, the distribution of the UI outcome revealed that 83% of patients rarely or never experienced UI at diagnosis and only 3% reported the most severe type, which is reported in the EPIC-26 questionnaire as “More than once a day”. However, as demonstrated in **Figure 1**, the severe type of UI was more frequent after treatment. The proportion of patients without UI decreased to 65% one year after diagnosis and to 63% after two years. This distribution of the UI outcome is imbalanced, with a larger proportion of patients having a negative outcome (no UI) and a smaller proportion having a positive outcome (UI).The association between a treatment and a UI outcome at each time point is provided in **Supplementary Table 3**.

In addition, univariate analysis found that the treatment choice is significantly associated (P<0.001) with UI. The majority of patients opting for active surveillance rarely or never experienced UI (79% and 74%), while those undergoing RP were more likely to experience UI (62% and 58%).

### Training and validation of models

**Figure 2:**
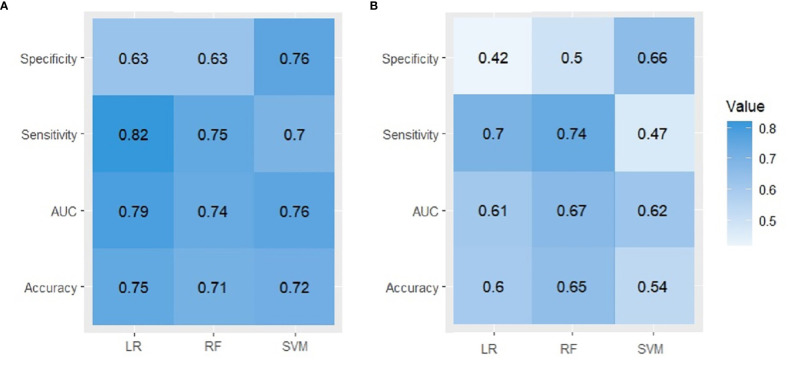
A comparison between observed and predicted UI derived from three ML algorithms in external validation. **(A)** first year, **(B)** second year.

**Figure 3:**
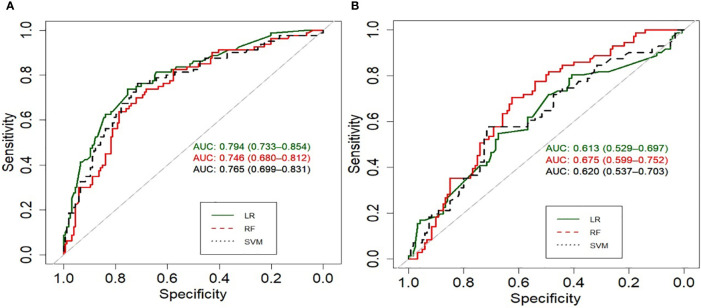
Receiver operating characteristic curves (ROC) of the models predicting UI at **(A)** first year, **(B)** second year.

**Figure 2** summarizes the performance of the models in predicting risk of UI on an test dataset. The LR model performed better in external validation than other models with an accuracy of 0.75, a sensitivity of 0.82, and an AUC of 0.79 (95% CI) for the first-year outcome. RF achieved the best performance with an accuracy of 0.65, an AUC of 0.67, and a sensitivity of 0.74 for the 2-year outcome. The accuracy of 2-year models varies from 0.65 for RF, 0.6 for LR to 0.54 for SVM, the ROC curves (**Figure 3**) indicate that the accuracy of different algorithms was approximately similar. SVM performed the best as reflected by its specificity (0.42 for LR vs. 0.5 for RF and 0.66 for SVM). Positive predictive value (PPV) and negative predictive value (NPV) of all models are presented in **Supplementary Table 4**. In addition we performed calibration of the original models for the 1-year and 2-year models (**Supplementary Figure 1**).

### Model explainability

In our study, we compared the interpretability of LR, RF, and SVM models with interaction terms. The LR models provided us with coefficients that indicate the relative importance of each feature in making predictions, making them more interpretable than the RF and SVM models which do not provide coefficients that are easy to interpret (**Supplementary Table 5**). Additionally, we generated a nomogram from the LR model which graphically represents how the model makes predictions. The nomogram allows us to easily visualize the impact of each feature on the predicted outcome. Nomogram was constructed to estimate the likelihood of developing UI based on the LR model for a one-year follow-up (**Supplementary Figure 2**).

### Important predictors

**Figure 4:**
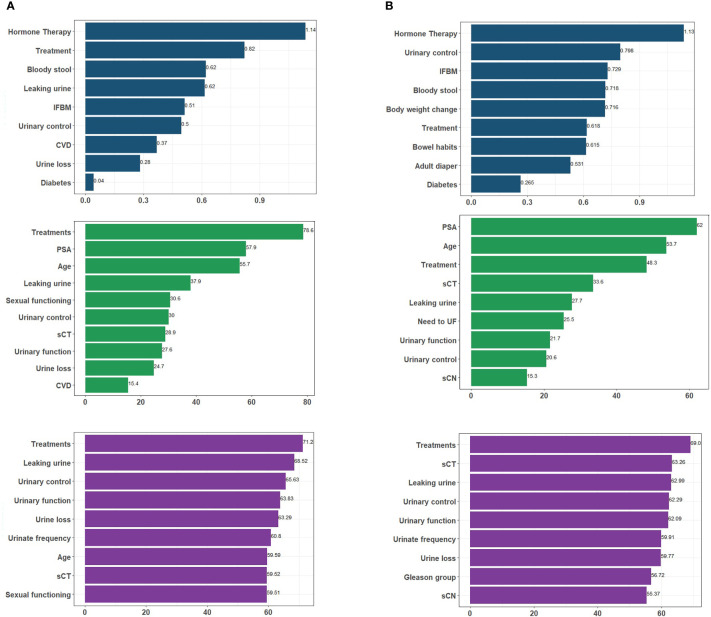
An overview of the most important variables selected by different algorithms, as well as their influence levels. Each color represents predictors of a particular algorithm, i.e. dark blue for LR, green for RF, and purple for SVM for **(A)** 1-year; **(B)** 2-year follow-up. Treatment. IFBM, Increased frequency of bowel movement; PSA, prostate-specific antigen; sCT, Tumor T stage; sCN, Tumor N stage; CVD, cardiovascular disease; UBM, urgency of have bowel movement; UF, urinate frequently.

Feature selection was performed prior to training with selected algorithms and nine variables were identified as important predictors of experiencing UI after treatment for each model except for the RF first-year model. Although the RF and SVM models are not inherently interpretable, feature importance metric can be used to gain some understanding of how the model makes predictions. Based on the influence of each feature across the six models, we can see the extent of influence each feature has on the models (**Figure 4**). Treatment group had the most impact on SVM and RF models and the second most for the LR at 1-year outcome. **Figure 4** shows the extent of influence each feature has on the models.

**Figure 5:**
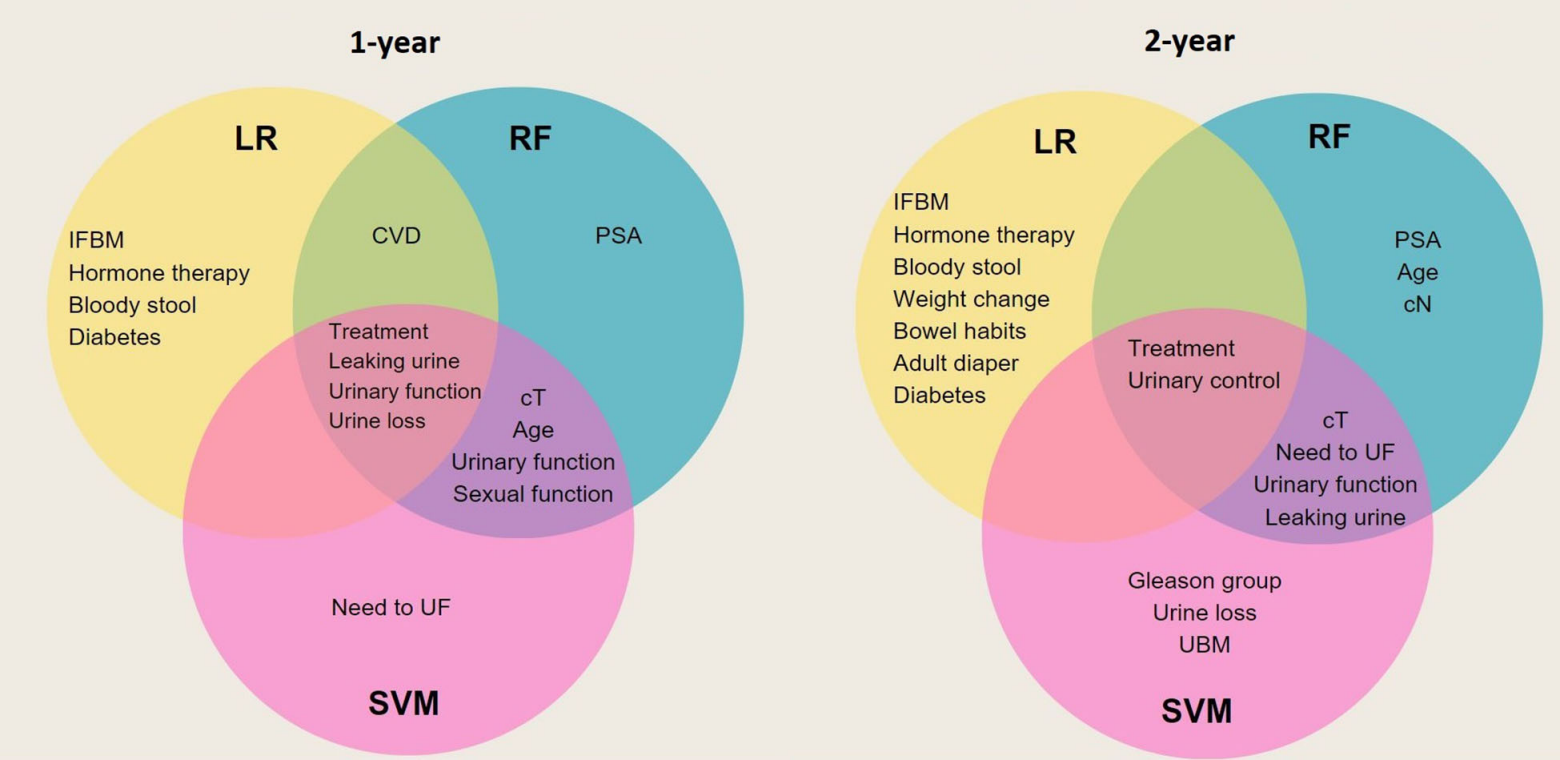
Venn diagram that illustrates overlap of predictors between generated models. Each circle represents the predictors of a specific model. Those predictors that are common between all three models are placed in the center overlap area. IFBM, Increased frequency of bowel movement; PSA, prostate-specific antigen; sCT, Tumor T stage; sCN, Tumor N stage; CVD, cardiovascular disease; UBM, urgency of have bowel movement; UF, urinate frequently.

As demonstrated in **Figure 5** most of the variables selected as predictors by LR, RF, and SVM are different from each other. Four baseline variables were found by all three algorithms for the 1-year follow-up: leaking urine (EPIC-26 question 4), urinary control (EPIC-26 question 2), treatment group (RB, EBRT, BT or AS), and urine loss (EPIC-26 question 1). For both time points, RF and SVM models had more predictors in common. Furthermore, the majority of predictors selected by algorithms were from PROMs, indicating the importance of pre-treatment conditions rather than clinical parameters in predicting treatment outcomes (**Supplementary Table 2**).

## Discussion

This study aimed to predict UI post-treatment in patients with localized prostate cancer using clinical, demographic, and pretreatment features. To this end, we developed and validated six different ML models according to TRIPOD type 3, to predict UI at 1-year and 2-year follow-up for men with localized prostate cancer. The external validation results indicated that the LR model performed best with an accuracy of 75%, a sensitivity of 82% and an AUC (CI:95%) of 79% in identifying patients at risk of developing UI 12 months after treatment. However, in terms of specificity SVM showed the best performance (76%). The performance of the RF model was lower compared to LR and only in terms of sensitivity was higher than SVM (75% vs. 70%). In contrast, none of the algorithms was successful in externally validating the 2-year outcome. The accuracy varied from 0.65 for RF, 0.6 for LR to 0.54 for SVM, and LR had the lowest specificity (0.42).

A few studies have investigated the possibility of predicting pre-treatment decisions related to UI. In the study conducted by Park et al., recovery from UI three months post-surgery was examined on retrospective data using five algorithms including k-nearest neighbor, decision tree, SVM, RF and LR. They used clinical, demographic and imaging data of 166 patients and the SVM algorithm yielded the best performance with an AUC of 0.65 (12). In comparison with our study, their sample size was smaller and they were, therefore, unable to perform an external validation according to TRIPOD type 3. While imaging data played a major role in their research, we focused on evaluating patients’ quality of life before and after treatment using PROMs data. As a result, there was no overlap among predictors. In another study, Laviana et al. developed a tool that predicted QoL outcomes six months to five years after treatments using demographics, PROMs, and clinical data of 2563 men with localized prostate cancer who received AS, EBRT, or RP. Their model achieved bias-corrected R-squared values of 0.232 for UI and found age, treatment, and baseline scores as the most important predictors. Compared to our dataset, their study included more patients for longer periods of time. However, their model was only internally validated (23). In the study conducted by Pinkhasov et al., the probability of experiencing UI was evaluated after the robot-assisted radical prostatectomy. Their dataset includes clinical and demographic data of 680 men and their LR model achieved AUCs between 0.64 and 0.80 across 24 months. No overlap in predictors was found and the study lacked external validation (24).

The predictors found in our study are clinically relevant, such as pretreatment (in)continence status, the type of treatment, and hormone therapy as an adjuvant treatment. For example, we found that men who undergo prostatectomy have a higher risk of experiencing UI compared to those patients who choose other options (25, 26). In addition, UI before the start of treatment was also selected as a predictor in our models in concordance with existing literature (27). Furthermore, hormone therapy (28) and the presence of diabetes and cardiovascular disease were also selected by the models in concordance with existing literature (29). Despite the role of obesity in the development of UI in prostate cancer patients, we were unable to explore whether this was the case in our study. This was due to the high proportion of missing values; we did not have sufficient data to explore BMI as a predictor. However, we were able to include diabetes as a comorbidity, which is known to be correlated with the incidence of obesity (30–32). While age was statistically significant in the 1-year model (p=3e-4), it was not indicated as a predictor for UI in our study. This was most likely due to the fact that the 60-75 age group dominated the dataset (n=618, 73%).

The importance of predictors in developing accurate predictive models for urinary incontinence cannot be overstated. Our study compared LR, RF, and SVM models and found that RF and SVM models showed more overlap in their performance than LR. While the accuracy of the three models was similar, interpretability is a crucial factor in clinical implementation (33). These three models have varying levels of explainability as simpler models like LR are often easier to interpret than more complex models like R (34) F. This makes LR advantageous in some applications such as healthcare where understanding how the model makes predictions is a priority (10). The coefficients provided by the LR model indicate the relative importance of each feature in making predictions, and the nomogram generated from the LR model allows for easy visualization of the impact of each feature on the predicted outcome. This interpretability can be particularly valuable in guiding treatment decisions, such as when considering alternative treatment modalities like radiotherapy (external/brachy), where surgery carries a higher risk of side effects On the other hand, RF and SVM models do not provide a straightforward way to visualize how the features impact the predicted outcome, which makes them less interpretable. Improving model interpretability was not the primary objective of our study; therefore we did not explore methods such as partial dependence plots or surrogate models to enhance interpretability (35).

Our study had some limitations. First, Our study was limited by the dataset size, which may have affected the comparison of ML algorithms, particularly RF and SVM, as they may require a larger number of samples to generate reliable predictions. Larger and more diverse datasets are needed to validate our results and provide more conclusive evidence. Secondly, while the second-year model achieved good accuracy in training, it had lower performance in the validation set. The lower number of data available for the 2-year models compared to the 1-year models, coupled with the longer time interval between predictors and outcomes, heightens the probability of intervening factors influencing the outcome beyond the baseline that were not assessed. For instance, certain patients who were at high risk of urinary incontinence might have received treatment for their condition, leading to potential bias in our data. This lack of accurate independent validation is a clear indicator that this model is not ready yet for clinical use. We suggest further investigations with a larger sample size and improved data quality control measures, such as reducing missing data or incorporating external data sources to mitigate potential bias. In addition, although UI is a less common side effect of radiation therapy (RT), it’s important to note that a significant proportion of patients with localized prostate cancer in our dataset underwent RP or AS, which may have resulted in less accurate predictions for the RT and BT treatment groups. Therefore, we suggest that others validate our model using their own RT or BT data to confirm its accuracy in these groups. Another limitation arose from the fact that the dataset was imbalanced toward the outcome. To solve this we trained data by performing upsampling. Finally, the current model was developed and validated using data from the Dutch population, so our results may not be generalizable to other populations outside the Netherlands.

## Conclusion

Our study aimed to develop prediction models for UI in men with localized prostate cancer using three different classifiers. The results showed that the LR algorithm outperformed the RF and SVM classifiers in predicting UI for a 1-year follow-up in an external dataset. These findings suggest that transparent and interpretable models can provide high performance to both patients and clinicians while meeting the transparency requirements for AI adoption. Consequently, we integrated the LR-based 1-year model into a prototype patient decision aid (PDA) to support SDM.

## Data availability statement

The datasets presented in this article are not readily available due to patient privacy. Requests to access the datasets should be directed to the Netherlands Cancer Registry, (https://iknl.nl/forms/dataapplication), and requests should be referred to by the following reference number: K19.117. All applications reviewed to ensure compliance with national privacy laws and the objectives of the Dutch Cancer Institute (IKNL). The code used to generate the results in our manuscript on GitHub (https://github.com/riannefijten/incontinenceArticle).

## Ethics statement

The studies involving human participants were reviewed and approved by METC azM/UM Maastricht university medical center (MUMC+). The patients/participants provided their written informed consent to participate in this study.

## Author contributions

RF: Conceptualization, Data Curation, Formal Analysis, Funding Acquisition, Investigation, Methodology, Project Administration, Resources, Software, Supervision, Validation, Visualization, Writing original Draft Preparation, Review and Editing. HH: Data Curation, Formal Analysis, Investigation, Methodology, Project Administration, Software, Visualization, Writing original Draft Preparation, Review and Editing. BO: Formal Analysis, Software, Visualization, Writing original Draft Preparation, Review and Editing. AD, IB: Formal Analysis, Supervision, Validation, Writing original Draft Preparation, Review and Editing. ZZ: Formal Analysis, Validation, Writing original Draft Preparation, Review and Editing. HP, LK, KA: Conceptualization, Data Curation, Writing original Draft Preparation, Review and Editing. BV, JR, RV, LH, EB: Validation, Writing original Draft Preparation, Review and Editing. All authors contributed to the article and approved the submitted version.
