# Supplementary material for: A comparison of machine learning models for predicting urinary incontinence in men with localized prostate cancer
Source: Front Oncol. 2023 Apr 12;13:1168219. doi: 10.3389/fonc.2023.1168219 (PMC10130634; doi:10.3389/fonc.2023.1168219)
Supplement: Supplementary file 1 [file DataSheet_1.docx]

# Supplementary

Table 1.The variables used as input for the prediction models for both years. The models in which they were used are depicted as well as the question of the EPIC questionnaire they apply to (if applicable). Additionally, the possible input values are described with a number and the meaning of that number as input. The numbers in the last column are numbers from the data.

| **Variable Name** | **Short description** | **Relates to EPIC-26 question** | **Input values** |
| --- | --- | --- | --- |
| treatments | The four big treatments groups |  | 1 = prostatectomy  2 = EBRT  3 = brachytherapy  4 = no active therapy |
| epic26_1_urineverlies | urine loss during last 4 weeks | EPIC-26 question 1 | 1 = > once a day  2 = once a day  3 = > once a week  4 = once a week  5 = rarely/never |
| epic26_2_urineophouden | urinary control | EPIC-26 question 2 | 1 = no control  2 = frequent dribbling  3 = occasional dribbling  4 = total control |
| epic26_3_verbanden | pads or adult diapers | EPIC-26 question 3 | 1 = None  2 = 1 pad per day  3 = 2 pads per day  4 = 3 or more pads per day |
| epic26_4_nadruppelen | dripping/leaking urine | EPIC-26 question 4a | 1 = no problem  2 = very small problem  3 = small problem  4 = moderate problem  5 = big problem |
| epic26_5_pijnplassen | pain/burning on urination | EPIC-26 question 4b | 1 = no problem  2 = very small problem  3 = small problem  4 = moderate problem  5 = big problem |
| epic26_6_bloedurine | bleeding with urination | EPIC-26 question 4c | 1 = no problem  2 = very small problem  3 = small problem  4 = moderate problem  5 = big problem |
| epic26_7_zwakkestraal | weak stream/incomplete emptying | EPIC-26 question 4d | 1 = no problem  2 = very small problem  3 = small problem  4 = moderate problem  5 = big problem |
| epic26_8_aandrang | need to urinate frequently | EPIC-26 question 4e | 1 = no problem  2 = very small problem  3 = small problem  4 = moderate problem  5 = big problem |
| epic26_9_urineprobleem | urinary function | EPIC-26 question 5 | 1 = no problem  2 = very small problem  3 = small problem  4 = moderate problem  5 = big problem |
| epic26_10_drangontlasting | urgency of have bowel movement | EPIC-26 question 6a | 1 = no problem  2 = very small problem  3 = small problem  4 = moderate problem  5 = big problem |
| epic26_11_vakerontlasting | increased frequency of bowel movements | EPIC-26 question 6b | 1 = no problem  2 = very small problem  3 = small problem  4 = moderate problem  5 = big problem |
| epic26_12_controledef | control loss of stool | EPIC-26 question 6c | 1 = no problem  2 = very small problem  3 = small problem  4 = moderate problem  5 = big problem |
| epic26_13_bloedontlasting | bloody stool | EPIC-26 question 6d | 1 = no problem  2 = very small problem  3 = small problem  4 = moderate problem  5 = big problem |
| epic26_14_krampdarm | abdominal/pelvic/rectal pain | EPIC-26 question 6e | 1 = no problem  2 = very small problem  3 = small problem  4 = moderate problem  5 = big problem |
| epic26_15_ontlastingprobleem | bowel habits | EPIC-26 question 7 | 1 = no problem  2 = very small problem  3 = small problem  4 = moderate problem  5 = big problem |
| epic26_16_goederectie | ability to have erection | EPIC-26 question 8a | 1 = very poor to none  2 = poor  3 = fair  4 = good  5 = very good |
| epic26_17_goedklaarkomen | ability to reach orgasm/climax | EPIC-26 question 8b | 1 = very poor to none  2 = poor  3 = fair  4 = good  5 = very good |
| epic26_18_kwalerectie | quality of erections | EPIC-26 question 9 | 1 = none at all  2 = not firm enough for sexual activity  3 = firm enough for masturbation/foreplay  4 = firm enough for intercourse |
| epic26_19_kwanterectie1 | frequency of erections | EPIC-26 question 10 | 1 = never when I wanted one  2 = <50% when I wanted one  3 = 50% when I wanted one  4 = >50% when I wanted one  5 = whenever I wanted one |
| epic26_20_oordeelseksfunc | ability to function sexually | EPIC-26 question 11 | 1 = very poor  2 = poor  3 = fair  4 = good  5 = very good |
| epic26_21_problseksfunc | sexual functioning, how big a problem | EPIC-26 question 12 | 1 = no problem  2 = very small problem  3 = small problem  4 = moderate problem  5 = big problem |
| epic26_22_opvliegers | hot flashes | EPIC-26 question 13a | 1 = no problem  2 = very small problem  3 = small problem  4 = moderate problem  5 = big problem |
| epic26_23_gevoeligeborsten | breast tenderness/enlargement | EPIC-26 question 13b | 1 = no problem  2 = very small problem  3 = small problem  4 = moderate problem  5 = big problem |
| epic26_24_depressie | feeling depressed | EPIC-26 question 13c | 1 = no problem  2 = very small problem  3 = small problem  4 = moderate problem  5 = big problem |
| epic26_25_weinigenergie | lack of energy | EPIC-26 question 13d | 1 = no problem  2 = very small problem  3 = small problem  4 = moderate problem  5 = big problem |
| epic26_26_gewicht | body weight change | EPIC-26 question 13e | 1 = no problem  2 = very small problem  3 = small problem  4 = moderate problem  5 = big problem |
| sCT | Tumor T stage |  | 1 = T1  2 = T2  3 = T3 |
| sCN | Tumor N stage |  | 0 = missing data  1 = N0  2 = X^a^ |
| nLeeft | Age *at diagnosis* |  | age in years |
| ch_indexgr | charlson comorbidity index simplified |  | 0 = missing data  1 = no comorbidities  2 = 1 point  3 = >=2 points |
| psa_diag | PSA (prostate-specific antigen) level at diagnosis |  | *ng/M* |
| gleason_group | Gleason group |  | 1 = group 1 (gleason score 6)  2 = group 2 (gleason score 3+4=7)  3 = group 3 (gleason score 4+3=7)  4 = group 4 (gleason score 4+4=7)  5 = group 5 (gleason score 9-10) |
| diabetes | The presence of diabetes |  | 1 = no diabetes  2 = diabetes |
| cardiovascularDisease | The presence of cardiovascular disease (CVD) |  | 1 = no CVD  2 = CVD |
| hormoneTherapy | Hormone therapy given to patients |  | 1 = no hormone therapy given  2 = hormone therapy given |
| alg_rook | Alcohol use |  | 1 = no  2 = previously  3 = yes |
| alg_alc | Smoking status |  | 1 = non smoker  2 = former smoker  3 = current smoker |

^a^ *N0 indicates that there are no cancerous lymph nodes in nearby areas*

Table 2. Multicollinearity assessment of statistically significant predictors

| **Predictor** | **VIF** |
| --- | --- |
| Diabetes | 1.035908 |
| Cardiovascular disease | 1.053124 |
| Urine loss during last 4 weeks | 1.673921 |
| Increased frequency of bowel movements1 | 1.082678 |
| Bloody stool | 1.070508 |
| Dripping/leaking urine | 1.548691 |
| Urinary control | 1.773658 |
| Treatments | 1.198442 |
| Hormone therapy | 1.026778 |

Abbreviation: VIF, variance inflation factor

Table 3. Distribution of UI in patients at diagnosis, 1-year and 2-year post diagnosis. For 1-year and 2-year the percentages are provided for each treatment separately. Explain abbreviations in footnote

|  | **Diagnosis (n=847)** | **1-year (n=847)** | | | | | **2-year (n=670)** | | | | |
| --- | --- | --- | --- | --- | --- | --- | --- | --- | --- | --- | --- |
|  |  |  | AS (n=350) | RP (n=279) | BT (n=166) | EBRT (n=52) |  | AS (n=270) | RP (n=225) | BT (n=133) | EBRT (n=42) |
| More than once a day | 25 (3%) | 111 (13%) | 17 (5%) | 87 (31%) | 4 (2%) | 3 (6%) | 79 (12%) | 18 (7%) | 57 (25%) | 4 (3%) | 0 (0%) |
| Once a day | 45 (5%) | 73 (9%) | 17 (5%) | 42 (15%) | 8 (5%) | 6 (12%) | 66 (10%) | 19 (7%) | 34 (15%) | 7 (5%) | 6 (14%) |
| More than once a week | 29 (3%) | 34 (4%) | 9 (3%) | 18 (6%) | 6(4%) | 1 (2%) | 33 (5%) | 10 (4%) | 18 (8%) | 4 (3%) | 1 (2%) |
| Once a week | 43 (5%) | 75 (9%) | 32 (9%) | 27 (10%) | 13 (8%) | 3 (6%) | 68 (10%) | 23 (9%) | 22 (10%) | 16 (12%) | 7 (17%) |
| Rarely or never | 705 (83%) | 554 (65%) | 275 (79%) | 105 (38%) | 135 (81%) | 39 (75%) | 424 (63%) | 200 (74%) | 94 (42%) | 102 (77%) | 28 (67%) |

Abbreviations: RP, radical prostatectomy; BT, brachytherapy; EBRT, external beam radiotherapy; AS, active surveillance

Figure 1: Calibration plots for 1-year and 2-year UI based on the different models (LR, RF, SVM) to visually represent the degree of similarity between the predicted probabilities and the observed frequencies. When a model is perfect, it conforms to the diagonal line. The plot indicates that all models had comparable performance in terms of predicting probabilities, with little variation between observed and predicted values.
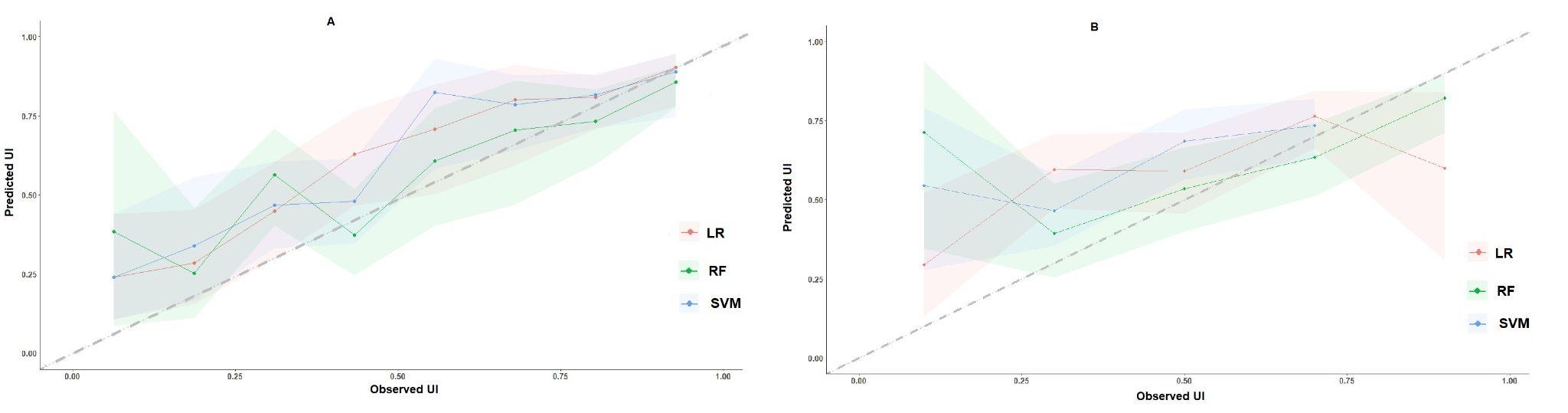


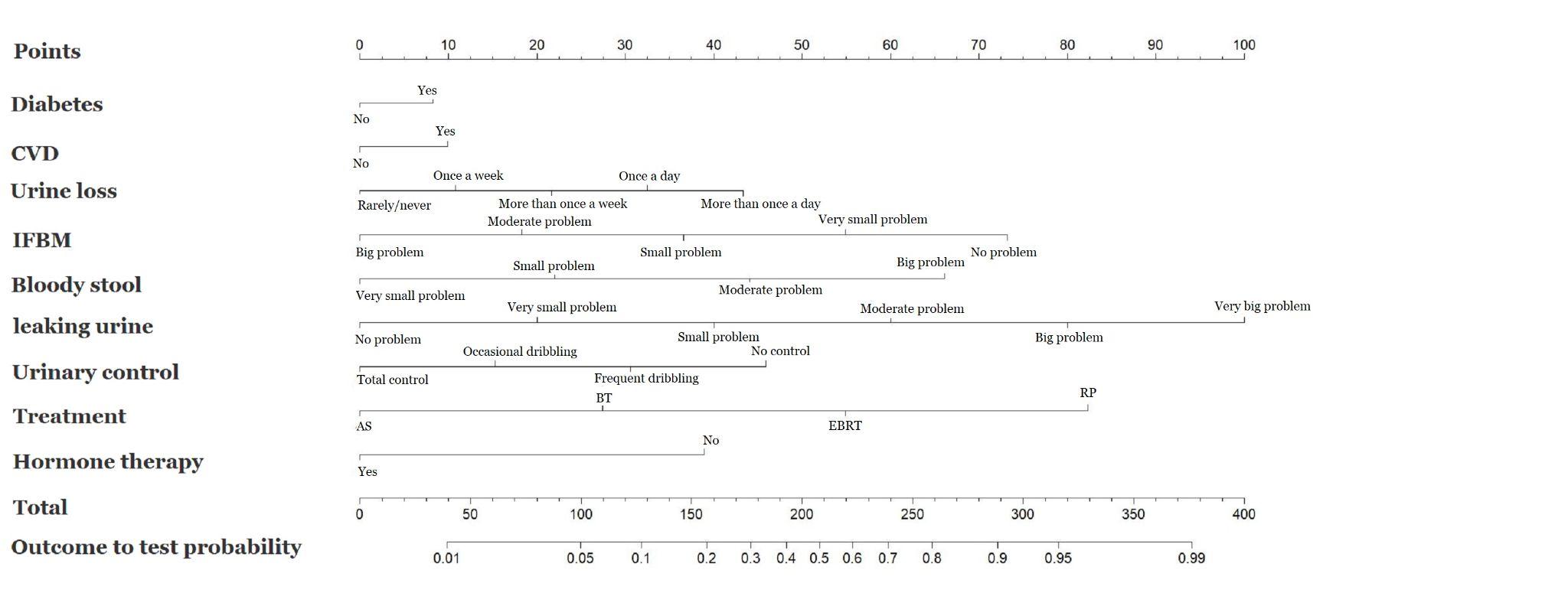


Figure 2. A nomogram for the 1-year model. A point score was assigned to each predictor, indicating its degree of influence on the likelihood of experiencing UI. By adding up the scores, the total point value can be obtained and used to calculate the probability of the outcome.

Table 4. Positive and negative predictive values of generated models

| Models | PPV | NPV |
| --- | --- | --- |
| LR-1year | 0.81 | 0.64 |
| RF-1year | 0.80 | 0.57 |
| SVM 1year | 0.85 | 0.57 |
| LR 2-year | 0.67 | 0.46 |
| RF 2-year | 0.71 | 0.53 |
| SVM 2-year | 0.70 | 0.42 |

Table 5. Model input variables and their logistic regression coefficients and P values.

| Variable | 1-year Coefficient | 1-year P value | 2-year coefficient | 2-year P value |
| --- | --- | --- | --- | --- |
| Hormone therapy | -1.136 | 0.001 | -1.136 | 0.003 |
| Treatment | -0.822 | <0.001 | -0.618 | <0.001 |
| Bloody stool | 0.622 | <0.001 | 0.718 | 0.034 |
| Leaking urine | 0.617 | <0.001 | — | — |
| Increased frequency of bowel movement | -0.513 | 0.001 | -0.728 | 0.001 |
| Urinary control | -0.496 | 0.014 | -0.797 | <0.001 |
| Cardiovascular disease | 0.368 | 0.038 | — | — |
| Urine loss | -0.282 | 0.029 | — | — |
| Diabetes | 0.042 | 0.875 | 0.264 | 0.396 |
| Weight change | — | — | -0.716 | 0.005 |
| Bowel habits | — | — | 0.615 | 0.004 |
| Adult diaper | — | — | 0.530 | 0.217 |
